# Review of Biological Activities of Some Rare Oils from Amazonian Plants

**DOI:** 10.3390/metabo15080554

**Published:** 2025-08-19

**Authors:** Luana Merckling-Almeida da Silva, Nicolas Merckling, Enrico Bigi, Katiane Cunha de Melo, Iuliana Popa

**Affiliations:** 1 Institut Médical de Champel, 1206 Geneva, Switzerland; dre-luana@docteur-merckling.ch; 2 Dumato Ltd.—Specialists in Amazonian Natural Products, 1260 Nyon, Switzerland; enrico.bigi@gmail.com; 3 Laboratório de Óleos Vegetais, Universidade Federal do Pará, Parque de Ciência e Tecnologia, Belém 66075-110, PA, Brazil; katianemelo@ufpa.br; 4 Pharmacy Department, University Paris-Saclay, Bâtiment Henri Moissan, 91400 Orsay, France

**Keywords:** biological activity, Amazonian plant oils, açai, andiroba, bacuri, buriti, cupuaçu, pracaxi, fatty acids

## Abstract

**Background/Objectives:** A great biodiversity of compounds from Brazilian medicinal plants are considered to be a promising source for biological activities in the cosmetics and pharmaceuticals sectors. Lipids and mainly fatty acids from Brazilian medicinal plants and their bioactive components are promising ingredients with proven diverse biological activities. To undertake a review of some rare oils of Brazilian medicinal plants with corroborated biological activities, we selected data from the scientific literature reporting the efficacy of plants used in folk medicine as antioxidant, anti-inflammatory or other types of activity of their oil phases extracts and isolated fatty acids. **Methods:** A search of the literature was undertaken by using the following web tools: Web of Science, SciFinder, Pub-Med and ScienceDirect. The terms “biological activity”, “anti-inflammatory”, “açai oil, andiroba oil, bacuri butter, buriti oil, cupuaçu butter and pracaxi oil”, and “free fatty acids” and “Amazonian plant oils” were used as keywords in the search engines. The Tropicos and Reflora websites were used to verify the origin of the plants, and only native plants from Brazil were included in this review. Only the publications reporting the use of well-accepted scientific protocols to corroborate the potential biological activities of rare oils of Amazonian plants were considered. **Results:** We selected 6 Brazilian medicinal plant oils extracts (acai, andiroba, bacuri, buriti, cupuaçu, pracaxi) with multiple biological activities. The observations were presented as a function of the oil origin and the most important biological activities were detailed. **Conclusions:** The collected data on the rare oils from Amazonian plants, in the form of crude extract and/or isolated compounds, showed significant biological activities involving different mechanisms of action, indicating that these oils could be an important source of lipids with biological activity.

## 1. Introduction

Historically, vegetable oils and fats have been—and continue to be—used in cosmetic, pharmaceutical and food preparations due to the presence of triglycerides and fatty acids. However, it is in the unsaponifiable part of the oils, which represents on average 2 to 4% of the total volume, that a whole pattern of bioactive components with the most diverse properties are concentrated. Several years ago, there were relatively few studies on rare oils from the Amazon. Nowadays, however, a growing number of studies are confirming some beneficial effects that were traditionally observed through empirical use by native Amazonian populations.

It is widely recognized that the Amazon rainforest is under pressure. The main factor leading to the deforestation and the loss of endemic species is agribusiness (mainly livestock and soy production) [1]. Amazonian vegetable oils are of interest as non-timber forest products with benefits for local communities with applications in traditional medicine, cosmetics and the food sector [2]. This could help to preserve the ecosystem and sustainably manage resources.

## 2. Methods

We ran a search in the literature by using the following web tools: Web of Science, SciFinder, Pub-Med and ScienceDirect. The terms “biological activity”, “antioxydant”, “anti-inflammatory” “wound-healing” “antimicrobial”, “antitumoral” “açai oil, andiroba oil, bacuri butter, buriti oil, cupuaçu butter and pracaxi oil”, “free fatty acids” and “Amazonian plant oils” were the most used keywords in the search engines. The Tropicos and Reflora websites were used to verify the origin of the plants, and only native plants from Brazil were included in this review.

We took into account only publications reporting the use of well-accepted scientific protocols to corroborate the potential biological activities of rare oils of Amazonian plants.

Our work focuses on 6 Amazonian oils extracted from açai, andiroba (also named crabwood), bacuri, buriti (also named aguaje or moriche), cupuaçu and pracaxi plants with multiple biological activities. In this respect, it highlights the diverse biological activities of Amazonian oils containing bioactive components such as antioxidant, anti-inflammatory, wound-healing, neuroprotective or antimicrobial activities.

For example, some of these plants’ extracts are already used in cosmetics, such as *Theobroma grandiflorum* seed butter (cupuaçu butter) in more than 920 cosmetic formulations, *Mauritia flexuosa* fruit oil (buriti oil) in 370 cosmetics, *Euterpe Oleracea* fruit oil (açai oil) in 320, *Carapa guaianensis* seed oil (andiroba oil) in 80 cosmetics, *Pentaclethra macroloba* seed oil (pracaxi oil) in 50 cosmetics, and *Platonia insignis* seed butter (bacuri butter) in about 24 references (according to a database that references about 130,000 commercialized cosmetic formulations) [3]. In comparison, more common oils such as almond oil, olive oil, and castor oil are each present in over 5000 formulations, whereas coconut oil, shea butter, and sunflower oil are each found in more than 15,000 formulations [3].

## 3. Lipid Composition of Some Rare Amazonian Oils

Beside the biological activities, this review explores the lipid profile of the Amazonian oils’ extracts for a better understanding of the biological properties that are investigated.

The composition of crude vegetable or fixed oils extracted from different parts of Amazonian plants largely depends on the methods used for extraction and refining. These oils typically consist of about 95% triacylglycerols, along with smaller quantities of free fatty acids, mono- and diacylglycerols, and variable levels of phospholipids, sterols (both free and esterified), triterpene alcohols, tocopherols, tocotrienols, phenolic substances, carotenes, chlorophylls, hydrocarbons, oxidation products, metals, and other trace components [4].

A comprehensive overview of the main fatty acids found in oilseed or oil pulps from Amazon rainforest plants is given in Table 1. The most abundant fatty acid found in seeds of Amazonian plants is omega-9 oleic acid [5,6], followed by palmitic acid (C16:0) [5], as evidenced in Table 1.

Compared to more common vegetable oils (such as olive oil, almond oil, sunflower oil, shea butter, castor oil, coconut oil) Table 2, the fatty acid fractions of cupuaçu and pracaxi contain long-chain saturated fatty acids (C20:0, C22:0 and C24:0). Otherwise, the Amazonian oils present a higher level of palmitic acid (C16:0) (60% in bacuri oil, to 25% in açai oil). In a lower amount than in the vegetable oils, the amazonian oils contain some linolenic (C18:2) but specifically palmitoleic (C16:1) as monounsaturated fatty acids. We can observe that only the pracaxi oil contains behenic fatty acid (C22:0), which is not observed in the most common vegetable oils. The particular composition of the Amazonian oils in palmitic, palmitoleic, oleic, and linoleic fatty acids (Table 1) is also responsible for the biological activities that we present in the second part of this review.

### 3.1. Lipids of Açai (Euterpe oleracea Mart.) Fruit Oil

**Figure 1:**
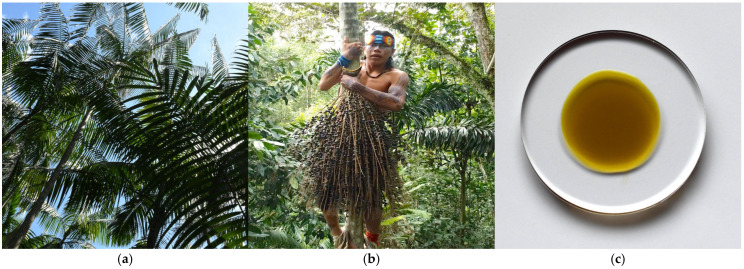
(**a**) The açai palm tree (*E. oleracea* Mart.) measures up to 20 m high; (**b**) the branch bearing açai berries is hand-harvested by climbing up the trunk (in the picture, a Shanenawa native young shaman picks açai berries); (**c**) the açai oil extracted from the pulp of the fruit is dark green (authors’ photographs).

The açai is a palm tree of the *Arecaceae* family whose trunk’s diameter is generally of less than 20 cm and whose height is between 3 and 25 m. It generally grows near mangroves and rivers in the north of the Amazon rainforest (Figure 1a). Its purple fruits resemble a bilberry, measuring 1.3 cm in diameter [11], and they are harvested twice per year (Figure 1b). The pulp is a highly nutritious source, owing to its high content of lipids (21–53%) and phenolic compounds. This explains the nomenclature of the species, “oleracea”. The açai berries are pressed to obtain a pulp, which is then dehydrated to obtain flour. The dehydrated flour is then pressed to obtain the oil. The color of the oil is dark green to violet (Figure 1c).

As described in Table 1, the predominant fatty acids include oleic acid (ranging from 54 to 75%), palmitic acid (22 to 30%) and linoleic acid (6 to 10%) [6]. Other fatty acids present include lauric acid, myristic acid, palmitoleic acid, stearic acid, vaccenic acid and linolenic acid [6,12,13,14].

At least five different sterols have been identified, with sitosterol being the most abundant (78%), followed by stigmasterol (6.5%), δ5-avenasterol (6.5%), campesterol (6.0%), and a smaller proportion of cholesterol (2.0%) [15].

Well known for its strong antioxidant capacity, primarily attributed to its high content of polyphenolic compounds, açai oil contains phenolic acids such as vanillic acid, syringic acid, p-hydroxybenzoic acid, protocatechuic acid, ferulic acid and gallic acid, all present at significantly higher concentrations compared to açai pulp [16]. The non-anthocyanin flavonoid profile includes various subclasses such as flavanols (e.g., catechin and epicatechin), flavones (such as orientin, homoorientin, and isovitexin), and flavonols (including rutin). Also present are procyanidin oligomers (dimers and trimers), dihydroflavonols (like dihydrokaempferol), methoxylated flavones (e.g., 5,4′-dihydroxy-7,3′,5′-trimethoxyflavone), and other compounds such as escoparin, apigenin, crisoeirol, velutin, and luteolin diglycoside [17,18].

### 3.2. Lipids of Andiroba (Carapa guianensis Aubl.) Seed Oil

**Figure 2:**
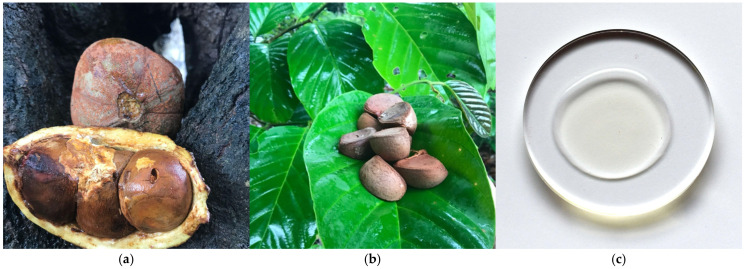
(**a**) Andiroba (*C. guianensis* Aubl.) woody fruit; (**b**) andiroba seeds from fruit; (**c**) andiroba oil extracted from the seed’s kernel has a pale-yellow hue (authors’ photographs).

The andiroba is a deciduous tree from the *Meliaceae* family. It is a large tree reaching 30 m in height. Known commercially as “Brazilian mahogany,” the wood is of economic importance, while the seeds (Figure 2a,b) serve to produce a yellowish oil with a pale hue (Figure 2c). The name derives from “*ãdi ‘roba*”, a Tupi term meaning “bitter oil”. The bitter taste of andiroba oil is linked to the presence of certain terpenes and limonoids [17]. The oil is extracted from the seeds that are first soaked for one week and then dried for two days before being crushed and pressed.

The main fatty acids are oleic acid (between 42 to 55%), palmitic acid (27% to 38%), linoleic acid (4 to 9%), and stearic acid (9 to 14%) [6,19,20,21]. Monounsaturated fatty acids make up approximately 50% of the total, while saturated and polyunsaturated fatty acids account for approximately 38 and 11%, respectively [4]. Thus, andiroba oil is abundant in essential fatty acids and contains non-lipid molecules such as triterpenes, tannins and alkaloids [5]. Wanzeler’s group [22] describes the presence of 4% monoolein and monopalmitin. There are also steroids and triterpenoids such as gedunin, 7-oxogedunine, deoxy-cericea-lactone or efusanin A [23]. Since 2013, different limonoids called carapanolides and carapanosins have been isolated from the oil of Carapa guianensis seeds [24].

### 3.3. Lipids of Bacuri (Platonia insignis Mart.) Seed Butter

**Figure 3:**
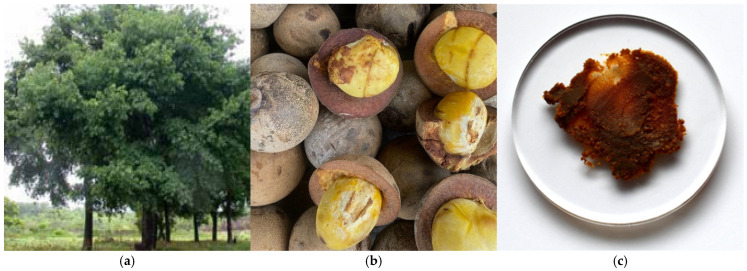
(**a**) Bacuri tree (*P. insignis* Mart.) measures up to 40 m high (Amazon oil—The rainforest company’s photograph); (**b**) when ripe, the fruit’s thick, leathery rind ranges from yellow to golden-brown and contains up to five seeds (authors’ photograph); (**c**) bacuri butter has a typical dark brown color (authors’ photograph).

The tree of bacuri, of the *Clusiaceae* family, is 30 to 40 m tall (Figure 3a). This deciduous tree only grows in the north of the Amazon. Its fruit, which weighs between 0.1 and 1 kg, is commonly consumed as juice. The industrial production of bacuri butter involves cold pressing of the fruit seeds (Figure 3b), yielding butter with a characteristic brownish color (Figure 3c).

Bacuri butter is solid at room temperature due to its high saturated fatty acid content. It contains an average of 64% saturated, 34% monounsaturated, and 2% polyunsaturated fatty acids (Table 1) [6]. The main triglyceride species are derived from palmitic (60%), oleic (28%) and palmitoleic (7%) acids [25,26]. Notably, tripalmitin relative abundance in bacuri butter is 20%. There are also small amounts of myristic, linoleic, lauric and stearic acids. In particular, 1,3-distearoyl-2-oleoylglycerol TG1 received extensive scientific attention due to its effective wound-healing ability. In addition, studies have reported the presence of xanthones (α-mangostin and γ-mangostin), polyisoprenylated benzophenones, such as garcinielliptone FC and dipertenes in bacuri seed extracts [27]. Moreover, four bioflavonoids, GB-2a, GB-1a, morelloflavone, and volkensiflavone, were also identified [27].

### 3.4. Lipids of Buriti (Mauritia flexuosa L.f.) Fruit Oil

Buriti oil has received attention in scientific research due to its bioactive properties and its various potential applications in nutrition, pharmacology and cosmetics.

**Figure 4:**
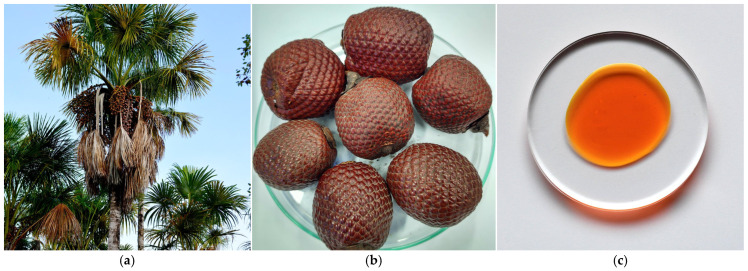
(**a**) Buriti palm tree (*M. flexuosa* L.f.) measures up to 35 m high; (**b**) buriti fruit is covered in overlapping, hard and shiny scales that protect the inner pulp; (**c**) buriti oil extracted from the pulp of the fruit has an intense orange hue, due to its high carotenoid content (authors’ photographs).

The buriti is a palm tree (*Aracecea* family), commonly referred to as moriche or aguaje. It generally grows in groups and can reach a height of 35 m (Figure 4a). The fruit is crushed to extract the pulp, which is fed into low-temperature ovens to remove the moisture (Figure 4b). The dried pulp, which has a flour-like consistency, is then poured into a hydraulic press to extract the oil (Figure 4c) [28]. Approximately 22 kg of fruit are required to produce 1 L of buriti oil, which contains oleic acid at concentrations ranging from 62 to 78% [7,29]. The fatty acid profile indicates that palmitic acid is the predominant saturated fatty acid, ranging from 16 to 26% [7,29]. Polyunsaturated fatty acids account for no more than 13.3% of the oil composition. Additionally, Speranza’s group [30] reported a triglyceride content of 93.33%.

Buriti oil has a very high value of carotenoids, mainly β-carotene, about five times more than in carrots. The Freitas group [31] noticed that in crude buriti oil, carotenoids are present in values between 1707 mg and 1730 mg/kg. They also highlighted the presence of 1041 mg/kg of tocopherol, mainly β-tocopherol (48%) and α-tocopherol (43%). There are also phenolic compounds (484 mg/kg) and flavonoids [29].

### 3.5. Lipids of Cupuaçu (Theobroma grandiflorum Willd.) Seed Butter

**Figure 5:**
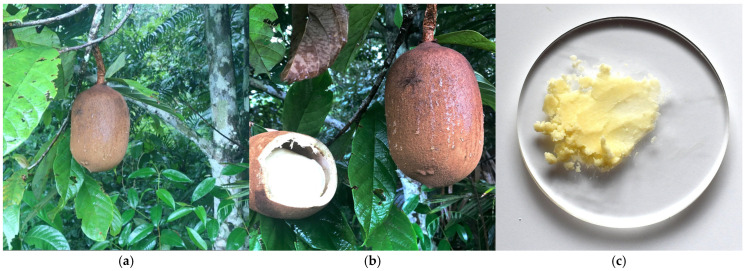
(**a**) Cupuaçu tree (*T. grandiflorum* Willd.) measures about 10 m high; (**b**) cupuaçu fruit on a branch with a cross-section view; (**c**) cupuaçu butter extracted from the seeds has a pale-yellow color (authors’ photographs).

Cupuaçu is a native and common tree throughout the Amazon. This tree from the *Malvaceae* family grows as a bushy tree from 5 to 15 m in height (Figure 5a). It is naturally cultivated within the jungle regions of northern Brazil, Peru, Colombia and Bolivia. The cupuaçu fruit is similar to the cacao fruit. It is an ovular-shaped fruit with brown peel surrounding the white pulp (Figure 5b). The pulp is commonly used for the production of juice or dessert. Cupuaçu butter is extracted through mechanical pressure from the seeds (Figure 5c).

Cupuaçu seeds are rich in fats, with fatty acids comprising approximately 60% of their dry weight. Its butter contains an average of 58% saturated fatty acids, 39% monounsaturated, and 3% polyunsaturated [32]. The most abundant fatty acids are oleic acid (40% to 46%) and stearic acid (33% to 36%) [7,33,34]. Compared to other Amazonian oils and butters, cupuaçu butter is notable for its higher levels of stearic acid and arachidic acid (7% to 12%). Other fatty acids present include palmitic acid (8%), linoleic acid and behenic acid (Table 1) [7]. Additionally, cupuaçu butter contains phytosterols, mainly β-sitosterol [34].

Phytochemical analyses show that both the pulp and seeds of cupuaçu are valuable sources of liposoluble antioxidant polyphenolic compounds, particularly flavones, flavan-3-ols, and proanthocyanidins [35,36]. Cupuaçu also contains proanthocyanidins mainly derived from epicatechin. The total of proanthocyanidin content in the seeds has been measured between 20 and 23 mg/g [35,37]. A similar composition was found by da Silva’s team [38], who detailed the presence of non-volatile organic compounds, such as over 17 flavonoids, 1 coumarin, 4 methylxanthines and 2 phenolic acids.

### 3.6. Lipids of Pracaxi (Pentaclethra macroloba (Willd.) Kuntze) Seed Oil

**Figure 6:**
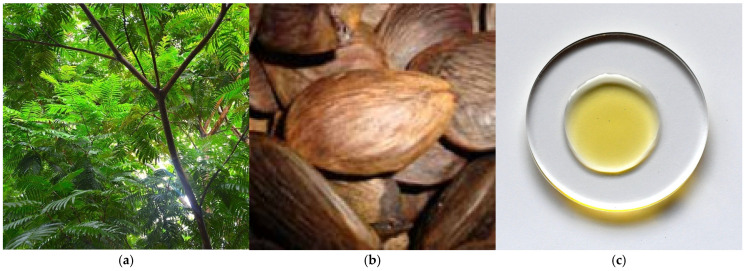
(**a**) Pracaxi tree (*P. macroloba* (Willd.) Kuntze) measures up to 25 m high (authors’ photograph); (**b**) pracaxi seeds grow in pods (Amazon oil—the rainforest company’s photograph); (**c**) pracaxi oil extracted from the seeds has a yellowish hue (authors’ photograph).

Pracaxi is a leguminous tree of the *Fabaceae* family that grows in northern Amazonia, as well as in southern Central America, and can reach a height of 25 m (Figure 6a). Its flat seeds grow in pods (Figure 6b). They are dried and then mechanically cold-pressed to extract the oil (Figure 6c). Oleic acid (47 to 53%), long-chain fatty acids and unsaturated fatty acids are predominant in pracaxi oil. It is the only oil with a high content of behenic acid (5 to 23%) and lignoceric acid (10 to 13%), giving it greater value than other vegetable oils [39,40,41], 16.1% [42] to 19.67% [5,43]. These differences may be inherent to plant varietal diversity and are often shaped by environmental factors such as climate and soil composition [5]. Several sterols have also been identified, with stigmasterol being the most abundant (53.96%), followed by β-sitosterol (33.96%) and campesterol (6.28%). The oil extracted from pracaxi is also rich in γ-tocopherol (416.13 mg/kg), δ-tocopherol (7.78 mg/kg), α-tocotrienol (93.53 mg/kg), and β-tocotrienol (79.92 mg/kg) [44].

## 4. Biological Activities of Some Rare Amazonian Oils and Butters

The biological activity of Amazonian oils is primarily attributed to their content of specific fatty acids, in addition to various lipid classes, some phospholipids and glycolipids, and a broad spectrum of minor bioactive components [45]. This section provides an overview of recent research on the specific biological activities of the previously discussed rare Amazonian oils and butters. A summarized overview of these activities is presented in Table 3.

### 4.1. Some Biological Activities of Açai (Euterpe oleracea Mart.) Fruit Oil

#### 4.1.1. Antioxidant Activity

Açaí oil is an important source of lipophilic antioxidants, which prevent lipid oxidation. Rufino’s group showed that it contains polyphenols 1.5 wt%, hydrolyzable tannins 1.6 wt% and condensed tannins 1.25 wt% [46]. They demonstrated in in vitro studies that the antioxidant capacity of açaí oil is higher (EC50 = 646.3 g/g DPPH (2,2-diphenyl-1-picrylhydrazyle)) than that of extra virgin olive oil (EC50 = 2057.27 g/g DPPH). Another in vitro study [16] showed that the phenolic components of açai oil, which are well known as antioxidants, remained largely stable during long-term storage at temperatures up to 40 °C, as well as after short-term heating at 170 °C for 10 min. These facts suggest a good stability of these compounds and their antioxidant properties.

De Almeida Magalhães’s group [47] demonstrated, by an in vitro study, the scavenging activity of the hydroxyl radical (OH) by 11.23%, 7.10%, 22.21%, and 8.5% in açai oil concentrations of 0.25, 0.5, 1.0, and 1.5 mg/mL respectively. The property does not appear to follow a dose-dependent pattern, given that the 1.0 mg/mL concentration yielded a considerably greater response than the others.

The dos Santos group [48] showed that the açai oil included at 2% daily in sheep diets under heat stress conditions enhanced antioxidant and anti-inflammatory activities in serum and milk, while increasing milk yield and quality, despite a decline in milk fat content.

Açai oil is widely used for healing and prevention of cutaneous disorders [11].

#### 4.1.2. Anti-Inflammatory Activity

A study from the Favacho team [13] showed an anti-inflammatory effect of açai oil in animal models (rats and mice) by oral administration for 6 days (1226.8 mg/kg). The carrageenan-induced edema was inhibited by 29.18%, the granulomatous tissue was inhibited by 36.66%, the ear erythema by 37.9% and the vascular permeability was reduced by 54.16%. In carrageenan-induced peritonitis, the neutrophils migration was reduced by 80.14% compared to the control group.

The same group [13] shows that fatty acids of açai oil have anti-inflammatory activity as good as fish and olive oils. They also observed antinociceptive effects of palmitic acid and anti-inflammatory activity of the oleic acid.

Another study [47] showed in an in vivo mouse model that açai oil reduced local inflammation by 37%, decreased edema volume, and lowered myeloperoxidase activity by around 42%. Among the phytosteroids present in the oil, β-sitosperol, stigmasterol, and campesterol are known to enhance cell metabolism and mitigate inflammatory responses. This property makes them popular ingredients in the cosmetics industry for preventing skin aging [50].

#### 4.1.3. Antitumoral Activity

Da Silva’s group [51] showed a cytotoxic effect of the oil (containing monoolein) in colorectal adenocarcinoma cells (CACO-2 and HCT-116) in an in vitro model by arresting the cell cycle. Moreover, they showed an increase in autophagy cellular processes proven by an increased expression of cellular marker LC3-B and Annexin A2 [51]. Due to these properties, monoolein is considered a promising candidate for research and therapeutic strategies targeting inflammatory diseases [132].

The Borges group [49] revealed antitumor effects of the oil (50 g/mL) by the inhibition of cell proliferation, migration, and colony formation in in vitro assessments on cervical cancer cell lines.

#### 4.1.4. Antimicrobial Activity

The oils from the seeds of the açaí palm showed an inhibitory effect in vitro against *Staphylococcus aureus* [54].

#### 4.1.5. Antihypercholesterolemic and Antihypertriglyceridemic Activity

Souza’s team [53] evaluated the effect of açai oil in an animal model (Wistar rats) with Triton-induced dyslipidemia. Their results have shown that the treatment with açai oil significantly improved the high-density lipoprotein (HDL) cholesterol concentration in the animals. This effect is likely attributable to the high levels of oleic and linoleic acids (unsaturated fatty acid) in açai oil. These fatty acids contribute to increasing HDL cholesterol, reducing triglycerides and total cholesterol levels, and promoting the removal of cholesterol from peripheral tissues by transporting it to the liver, which can help improve low-density lipoprotein cholesterol (LDL) profiles.

#### 4.1.6. Other Biological Activities

Laurindo’s group noticed other activities from the studied açai pulp such as neuroprotective, cardioprotective, hepatoprotective, renoprotective, antihypertensive, musculoskeletal health, antidiabetic, antileishmanial, and anticonvulsant activities [133].

### 4.2. Some Biological Activities of Andiroba (Carapa guianensis Aubl.) Seed Oil

#### 4.2.1. Antioxidant Activity

The linoleic acid content was related to the antioxidant potential of andiroba oil [19]. The Araujo-Lima group [4] compared the in vitro antioxidant activity of andiroba oil obtained through different extraction methods. The highest radical scavenging activity was observed in the oil extracted by cold-pressing dried seeds, where no heat was applied and processing time was minimized, suggesting that this method preserves bioactive compounds responsible for antioxidant activity.

#### 4.2.2. Anti-Inflammatory and Tissue-Healing Activity

Ribeiro’s team [25] emphasized that the andiroba seed lipid extract is traditionally used by Amazonian people for its anti-inflammatory effects, including muscle relaxation, joint pain relief, disinfection, and skin wound healing [62].

In a review on the anti-inflammatory properties of andiroba oil, the Fonseca group [56] reported that the oil reduces inflammation and promotes wound healing by modulating immune responses and enhancing processes such as phagocytosis, fibroblast activation, reepithelialization, and angiogenesis. Moreover, several formulations such as nanoemulsions, films, and gels demonstrated greater efficacy than the unprocessed andiroba oil in controlling inflammation and accelerating tissue repair in animal models [56].

The presence of monoolein as a biocompatible lipid molecule with potential applications in bone repair and drug delivery was described in andiroba oil. [22]. It exhibited anti-inflammatory activity in an animal study in golden Syrian hamsters by suppressing the immune response induced by lipopolysaccharide. Its mechanism of action involves reducing the production of pro-inflammatory mediators, including IL-12 p40, IL-6, TNF-α, and nitric oxide.

In an animal model using Wistar rats, a recovery of the anterior tibia muscle was shown after effort by treatment with andiroba oil and LED (light-emitting diode), and it confirmed a reduction in the oxidative stress and muscle damage [63].

#### 4.2.3. Antitumoral Activity

Andiroba oil showed antitumoral activity by inducing apoptosis in different cell lines in vitro [64,65,66].

Another group observed that andiroba oil induces apoptosis in gastric adenocarcinoma cells in vitro, demonstrating significant cytotoxicity without mutagenic effects, which suggests its potential as a candidate for therapeutic use. The apoptotic process is mediated by the fatty acids of the oil, which alter cell membrane integrity and activate programmed cell death pathways [67].

#### 4.2.4. Antimicrobial Activity

The Silva team [71] reviewed the effects of andiroba oil on living organisms in vitro. They pointed out that the oil was effective against *Xanthomonas axonopodis* bacteria. Mitigated antimicrobial activity was obtained on *Escherichia coli* and *Staphylococcus aureus*, low antimicrobial activity was found on *Klebsiella pneumoniae*, and no activity was found against *Enterococcus faecalis* and *Salmonella typhi*. Studies have also confirmed the antifungal activity of andiroba oil through its inhibitory effects on *Colletotrichum gloeosporioide*, *Sclerotium rolfsii*, *Postia placenta*, and *Trametes versicolor* [71].

The bactericidal as well as the antifungal activities in animals and humans of the oil extract from andiroba seeds were related to the fact that it contains mainly gedunin-type limonoids [4,55]. Other groups showed that the antimicrobial activity of andiroba seed oil was due to the presence of gedunine, tetranorthriterpenoids such as 6α-acetoxygedunine, 7-deacetoxy-7-oxogedunine, andirobin, and methyl angolensate [72,73].

#### 4.2.5. Antiparasitic Activity

Andiroba oil exhibits strong larvicidal activity against *Aedes aegypti*, the mosquito vector of dengue fever [75,76]. It also showed acaricidal effects against larvae of various tick species [71]. Additionally, andiroba oil and its limonoid-rich fractions have demonstrated antiplasmodial and anti-malarial activity against *Plasmodium falciparum* in vitro [71].

#### 4.2.6. Other Main Biological Activities

It was reported that andiroba oil is used by indigenous Amazonian and the Caboclos (mixed people who are living in the forest) to alleviate coughs and convulsions, heal wounds and bruises, treat skin diseases, arthritis, rheumatism, and ear infections, and serve as an insect repellent [5].

In the review of the group of Machado [73], they reported several other biological properties for the seed oil from andiroba used in folk medicine, such as treating fever, anti-allergic, analgesic and chemotherapeutic effects [68,69,105], and effectiveness against arthritis [134]. As for the group of Ribeiro [25], they reported that andiroba oil has proven properties as an insect repellent [78], in controlling mites [135], and in fighting intestinal sepsis [136], and it is used in lotions, shampoos, creams for hair, and soaps in the cosmetics industry [21].

Additionally, the content of linoleic acid was linked to cholesterol reduction and blood pressure regulation, and it is regarded as helpful in cancer prevention [20,21,73].

### 4.3. Some Biological Activities of Bacuri (Platonia insignis Mart.) Seed Butter

#### 4.3.1. Antioxidant Activity

Bacuri butter shows notable antioxidant potential, largely attributed to garcinielliptone FC (GFC). In vitro studies by Costa Junior et al. [94,95] demonstrated GFC’s ability to prevent lipid peroxidation and scavenge nitric oxide and hydroxyl radicals. Additional compounds such as α-mangostin and γ-mangostin [96] and 2-oleyl-1,3-dipalmitoyl-glycerol [97] may also contribute to the antioxidant activity.

The antioxidant activity of a xanthone-rich bacuri seed extract was demonstrated in a rat model. Reduced lipid peroxidation level and nitrite content in rat striatum was confirmed when they were pre-treated with 0.1 to 10 mg/kg of the extract, indicating that it has a protective effect on the central nervous system [98].

In a preliminary stage of his study on bioactive biflavonoids from bacuri, Ribeiro’s group [27] investigated the antioxidant effect of bacuri seeds extracts in vitro, obtained with three different extracting agents (hexane, ethyl acetate, and methanol). The ethyl acetate seeds extract presented the best antioxidant activity.

The Coêlho group [99] produced solid nanoparticles from bacuri butter and administered them in vivo to *Zophobas morio* larvae. Evaluation of reduced glutathione, nitrite, and myeloperoxidase levels confirmed the antioxidant activity of the formulation.

#### 4.3.2. Anti-Inflammatory Activity

Lustosa’s team [101] evaluated the anti-inflammatory effect of orally administered bacuri butter in a rat model with paw edema. All tested doses reduced inflammation within 4 h, with the highest dose (400 mg/kg) showing a significant effect by hour 5. In a second test, a 5% bacuri butter topical formulation also inhibited edema when applied before induction, showing comparable efficacy to 0.1% dexamethasone acetate, suggesting modulation of inflammatory mediators.

#### 4.3.3. Wound-Healing Activity

In traditional medical practices, seed butter extracted from bacuri was applied to treat wounds, eczema, herpes, and various other skin conditions. It was reported that tripalmitin has humectant characteristics and is present in a relatively high amount in bacuri butter. This triglyceride provides high penetration into the skin with wound healing properties [88,92].

The Dos Santos group [89] evaluated the wound-healing effects of a bacuri seed cream in Wistar rats. Chronic inflammation was observed on days 4, 7, and 14 after wound induction. By day 7, treated rats showed significant reepithelization compared to untreated controls.

The Mendes group [137] formulated creams with 5–15% of 1,3-distearoyl-2-oleoylglycerol (TG1) isolated from bacuri seeds and evaluated their wound-healing efficacy in Wistar rats with induced skin lesions. Histological analysis revealed enhanced fibroblast proliferation, angiogenesis, and reepithelialization, with improved wound retraction at all tested doses compared to the TG1-free control. In a separate study, Feitosa’s team [138] reported that TG1 exhibited low oral toxicity in rats, with no behavioral, hematological, or liver histological changes even at an acute dose of 30 mg/kg.

#### 4.3.4. Cardio-Protective Activity

In vivo studies using a hamster animal model of diet-induced hypercholesterolemia were conducted to assess the cardioprotective effects of bacuri butter [91]. Bacuri butter, administered at 25 and 50 mg/kg/day, reduced atherosclerosis risk by lowering the atherogenic index, coronary risk index, and LDL/total cholesterol ratio, while raising the HDL ratio. These effects were attributed to the presence of unsaturated fatty acids, xanthones, and prenylated benzophenones.

#### 4.3.5. Vasorelaxant Activity

Arcanjo’s group [26] investigated the vasorelaxant properties of garcinielliptone FC (GFC) using an ex vivo model of rat mesenteric artery rings. GFC induced endothelium-independent relaxation in vessels pre-contracted with phenylephrine but had no effect on KCl-induced contraction. The findings of their study indicate that GFC promotes vasorelaxation by reducing calcium influx and mobilization, thereby limiting smooth muscle contraction.

#### 4.3.6. Immunomodulatory Activity

The Lustosa group [104] investigated the immunomodulatory effects of a hexane extract from bacuri seeds through in vitro assays. The extract was tested on murine peritoneal macrophages by evaluating phagocytic activity, lysosomal volume, and nitrite production—a proxy for nitric oxide-mediated cytotoxic activity. All parameters were measured colorimetrically and showed a concentration-dependent immunostimulatory effect. At 12.5 µg/mL, the extract increased macrophage phagocytic capacity by 49.8% and lysosomal volume by 9.2%. The highest nitrite production (12.4% increase) was observed at 100 µg/mL. These findings were supported by in vitro and in vivo toxicological assessments using a Wistar rat animal model, which indicated low toxicity, minimal hemolytic activity, and no observed clinical or behavioral changes following acute oral administration at 2 g/kg.

#### 4.3.7. Antiparasitic Activity

Silva’s group [105] reported the in vitro effect of garcinielliptone FC (GFC) isolated from bacuri seeds against the blood flatworm *Schistosoma mansoni*. GFC (50 µM) was able to kill all *Schistosoma mansoni* adult worms within 24 h. It was demonstrated that GFC induced tegumental damages in the worms and altered their reproductive fitness at sub-lethal concentrations (<3.125 μM). Furthermore, no cytotoxicity was observed towards mammalian cell lines, even at the highest tested concentration (50 µM), indicating that GFC is a good antiparasitic candidate.

Bacuri seed extracts have shown efficacy against *Leishmania amazonensis*, the protozoan responsible for cutaneous leishmaniasis. The Costa Junior group’s studies [95,100] demonstrated the in vitro cytotoxic and leishmanicidal activity of GFC. Coêlho’s group [93] formulated emulgels combining bacuri butter with 1–3% amphotericin B to reduce the drug’s toxicity while enabling topical delivery instead of the standard parenteral route administration; in vitro tests suggested synergistic antileishmanial effects. Lustosa’s group [92] reported that a hexane extract reduced infection rates in murine macrophages in a dose-dependent manner and was 18 times more selective for parasites than host cells. These findings were supported by in vivo studies in a mouse animal model, where topical treatments with 5% bacuri butter significantly slowed ulcer progression compared to controls [88].

#### 4.3.8. Central Nervous System Stimulator

The Da Silva team [102] investigated the effect of GFC when administered at doses of 25, 50 and 75 mg/kg on the central nervous system on a mouse animal model subject to pilocarpine-induced status epilepticus. An increased latency time to first seizure for each tested dose was observed when compared with seized mice, as well as a decrease in mortality rate. This suggests that GFC may have anticonvulsant properties and can decrease the frequency of status epilepticus onset. Evaluation of the mice’s hippocampus showed that GFC modulated the γ-amino butyric acid content (which is involved in excitatory action and inhibitory action) and the glutamate content (which is involved in excitatory action). In addition, acetylcholinesterase enzyme’s activity was increased. It is thought that GFC may be a useful bioactive molecule to produce neuronal protection.

#### 4.3.9. Gastroprotective

Bacuri seed butter and extract show potential in treating peptic ulcers due to their antioxidant properties. To enhance bioavailability, Lima-Nascimento’s group [103] developed a β-cyclodextrin inclusion complex with bacuri seed extract. In vivo tests on ethanol-induced gastric lesions in a mouse animal model showed an 89.5% reduction in ulcer area with 100 mg/kg of the complex containing 30% of extract. Similarly, the complex showed significant gastroprotective effects in a rat model of ischemia-induced lesions. The activity is attributed to antioxidant compounds like xanthones and benzophenones of bacuri seeds extract.

### 4.4. Some Biological Activities of Buriti (Mauritia flexuosa L.f.) Fruit Oil

#### 4.4.1. Antioxidant Activity

Buriti fruit oil is high in carotenoids, especially β-carotene, and has vitamin E isomers such as α-tocopherol and β-tocopherol. These vitamins are associated with antioxidant activities. Its high concentration of carotenoids and tocopherols has an ability to neutralize free radicals, which helps prevent oxidative stress and chronic diseases, as demonstrated in tests such as Oxygen Radical Absorbance Capacity (ORAC) and Ferric Reducing Antioxidant Power (FRAP) [106].

#### 4.4.2. Anti-Inflammatory Activity

In addition, the bioactive compounds present in the oil, such as carotenoids and phytosterols, are effective in reducing inflammatory processes, with studies suggesting that the oil can strengthen the immune system and modulate inflammatory responses. The Ferreira group [112] highlighted that buriti oil scavenges hydroxyl radicals produced by activated leukocytes at injury sites during inflammatory responses. Additionally, the oil inhibits nitric oxide production, thereby helping to reduce inflammation.

#### 4.4.3. Healing Activity

Furthermore, in studies comparing the chitosan gel to buriti oil in an in vivo mouse model [112], the Amazonian buriti oil showed an acceleration of the healing process in skin lesions, promoting fibroblast proliferation, collagen fiber synthesis, and skin re-epithelialization.

#### 4.4.4. Photoprotective Activity

Buriti oil is widely used in cosmetics due to its emollient properties, which promote moisturization and protection against sun damage. Buriti oil increases the cell viability of UVA/UVB irradiated cells, as Zanatta’s group [139] showed in their in vitro model with human keratinocyte cells and mouse embryonic fibroblast cells. This photoprotective activity is due to the carotenoids from buriti that are able to protect cells against photooxidative damages in skin. Parente’s group [113] demonstrated with a spectrophotometric measurement that when used alone, buriti oil offers a lower level of protection, being classified as low-protection, which can be used by people with darker skin. In this case, the oil could be used by these people as a natural photo-protector, absorbing UV radiation, and after that for downregulating the damages in skin very quickly. Otherwise, the buriti oil improves the sensory characteristics and the stability of cosmetic formulas.

#### 4.4.5. Antimicrobial Activity

With regard to antimicrobial activity, buriti oil showed in an in vitro model efficacy against various pathogenic bacteria, including *Escherichia coli*, *Pseudomonas aeruginosa*, *Klebsiella pneumoniae*, and *Staphylococcus aureus*, functioning as a natural antimicrobial agent that reduces the bacterial load in food and can be used in therapeutic applications, as reported by the teams of Leão [106], Morais [107], Ferreira [112] and Castro [114].

#### 4.4.6. Other Biological Activities

Finally, both buriti oil and flour showed potential for different activities by ingestion. In their review, Barboza’s group [29] highlighted other potential effects, such as antifungal, antithrombotic, anti-proliferative, antidiabetic, and prebiotic action as buriti oil modulates the intestinal microbiota, indicating positive implications for digestive health. There are also estrogenic and antiandrogenic activities, as shown by the Miyasaka group [115].

### 4.5. Some Biological Activities of Cupuaçu (Theobroma grandiflorum Willd.) Seed Butter

#### 4.5.1. Antioxidant Activity

Several studies investigated the antioxidant activities of polyphenols and flavonoids contained in cupuaçu butter, indicating that they trigger potential health benefits [35,36,122,123]. For example, Yang’s group [36] pointed out the biological activity of nine flavonoid antioxidants and two new sulfated flavonoid glycosides, theograndins I and II. Interestingly, theograndins from cupuaçu seeds exhibit higher water solubility in vitro, which may enhance their bioavailability compared to other flavonoids.

Pugliese’s group [35] evaluated in vitro the antioxidant activity of flavonoids and proanthocyanidins from fresh seeds versus ascorbic acid. The content of proanthocyanidin oligomers in seeds was about 23 mg/g, mainly of the epicatechin type.

Pinent’s group showed that a treatment with natural proanthocyanidin-rich extracts presents an antioxidant effect on absorptive cells and enterohormone-secreting cells of the gastrointestinal tract in an in vitro cell model and in vivo in animal study [122]. The effects of the treatment are dose- and time-dependent. The animal study revealed that a 25 mg/kg body weight could counteract the intestinal reactive oxygen species (ROS) when associated with a fasted condition.

In another study [123], it was mentioned that the concentrations of phenolic compounds increased during in vitro gastrointestinal digestion, with bioaccessibility reaching as high as 274.13% for total phenolics.

#### 4.5.2. Wound-Healing Activity

Due to its high content of oleic acid, cupuaçu butter is a valuable candidate for wound healing ability [126]. The wound healing properties of cupuaçu butter were investigated in an in vitro model on human skin fibroblasts cells [116,117]. Sano’s group [116] showed that treating in vitro cultures of fibroblasts with cupuaçu butter at a concentration between 0,1 and 10 μg/mL for 24 h showed a significant proliferative effect relative to control. The cupuaçu butter showed improvement in cell migration and wound healing in a scratch test. Also, the study showed that cupuaçu butter impacts the way in which growth factor genes are expressed in dermal fibroblast cells. Similar results were confirmed by the Barbalho group’s study [117], underlining that cupuaçu induces a gene expression pattern associated with tissue repair in a primary human dermal fibroblast model. Cupuaçu contributes to the reestablishment of a functional skin extracellular matrix in favor of skin elasticity and post-lesion hydration in a burned skin model [117].

#### 4.5.3. Emollient Activity

Cupuaçu seed butter is an excellent emollient. It restores the elasticity of the skin and provides good hydration [36]. The Fleck group [119] underlined cupuaçu butter’s transdermal penetration ability, providing antioxidants and hydration to the skin at the same time.

#### 4.5.4. Anti-Neurodegenerative Activity

Yanes’ group [121] studied the inhibition of acetylcholinesterase enzymes by cupuaçu fatty acids in vitro. Acetylcholinesterase enzyme is known to have a biochemical importance in humans, but its sudden increase may induce neurodegenerative conditions like Alzheimer’s disease. This study reported a moderate inhibition of about 40–48% in acetylcholinesterase [121]. An animal study suggests a promising potential for the use of polyphenols from Theobroma species used as complementary treatment in depression and stress management and resilience enhancement [118].

#### 4.5.5. Photo-Protective Activity

The Aparicio-Álvarez team [120] developed a stable emulgel-type sunscreen product containing up to 20% of cupuaçu butter. They used an in vitro study on a mouse model irradiated with UV light (UVB, 5000 J/m^2^, for 15 consecutive sessions of 30 min each). They showed that the sun protection factor (SPF) values of the emulgels with 5, 10, and 20% cupuaçu seed butter were 9.44, 9.63, and 11.67, respectively. The treated mice showed less damage and significantly reduced severity, comparable to a commercial standard photoprotector.

#### 4.5.6. Antitumoral Activity

A study [36] evaluated the cytotoxicity of two sulfated flavonoid glycosides and found that theograndin II, extracted from cupuaçu seed, exhibited moderate cytotoxic effects on human colon cancer cell lines in vitro.

#### 4.5.7. Antidiabetic Activity

The phenolic content in cupuaçu seeds has attracted attention for its potential anti-diabetic properties, particularly due to their ability to inhibit α-amylase, the enzyme responsible for breaking down complex carbohydrates like starch into simpler sugars such as maltose and glucose. The Andrade group [123] showed in vitro that cupuaçu seed butter inhibits 97% of α-amylase, which is promising for type 2 diabetes. Another finding of this research was that, following simulated gastrointestinal digestion under in vitro conditions, the aqueous seed extract retained flavonoids such as epicatechin, rhamnetin, and daidzein.

#### 4.5.8. Microbiological Activity

Bastos’ team [124] demonstrated that cupuaçu oily seed extract exhibits specific activity against *Plasmodium falciparum* in vitro, the parasite responsible for malaria. The cupuaçu extract inhibited *P. falciparum* growth by 20–24%.

### 4.6. Some Biological Activities of Pracaxi (Pentaclethra macroloba (Willd.) Kuntze) Seed Oil

The Amazonian population empirically used the oil and extract of seeds of pracaxi medicinally for different treatments such as erysipelas, wounds on skin, and snake bites, as well as for dysentery and diarrhea, and also as a therapeutic agent for the treatment of muscle pain and inflammation [40].

#### 4.6.1. Antioxidant Activity

Texeira’s group [125] isolated phenolic compounds from 31.92 to 54.05 mg GAE (gallic acid equivalents)/kg oil. The study highlighted a significant antioxidant activity with 21.81 to 41.03% inhibition of DPPH according to the samples. Recently, Eberhart’s team [41] found a higher phenolic compound: 67.43 mg GAE/kg oil. The Serra group [108] noticed that pracaxi oil has a high value of tocopherol (597.36 ppm) and some carotene, both well-known for their antioxidant activity.

#### 4.6.2. Anti-Inflammatory Activity

Simmons’ group [129] reported the use of a compounded medication containing 2% mupirocin (antibacterial agent), formulated in an anhydrous silicone base with pracaxi oil. In a human in vivo model, the preparation was applied topically to the ulcer three times daily. After six days of treatment, the ulcer had fully closed, and the patient noted a substantial reduction in pain. The reason for fast ulcer healing could be due to the high amounts of oleic, linoleic, and behenic acids, which have positive impacts on skin structure and permeability. In another study in an in vivo mouse model [126], these fatty acids also showed enhanced wound closure and improved healing by modulation of the inflammatory phase along with the downregulation in cell migration (CD3 + T lymphocytes, NK cells, and B lymphocytes) in and around the wound.

#### 4.6.3. Wound-Healing Activity

Pracaxi oil is used empirically by several indigenous Amazonian communities for health purposes, including the treatment of ulcers and bacterial infections [5,130].

In the clinical study of Banov’s group [43], a topical base containing pracaxi oil as an ingredient was used for wound healing. This pracaxi oil base and final ointments contained either 1% pentoxifylline (antifibrogenic activity), 1% caffeine (to promote circulation), 1% tranilast (as collagen synthesis inhibitor), or 2% mupirocin (as antibacterial). The study showed a real improvement in the wound scars (ankle, face or elbows) after 3 weeks with the base ointment treatment not containing any of these actives. Otherwise, the topical form, besides pracaxi oil, contained a significant amount of silicones and butylated hydroxy-toluene as solvent, which are not ideal for skin and wound healing. Another study confirmed the improvement in ulceration and wound healing after 30 days in a diabetic person when they use a similar topical application containing a pracaxi oil base [129].

These data suggest that pracaxi oil may be an excellent option for wound healing due to its fatty acid content, which plays a crucial role in maintaining cell membrane integrity and protecting the skin by preventing dehydration [5,140].

#### 4.6.4. Antimicrobial Activity

The Leal group [130], in their in vitro study on pracaxi oil’s antimicrobial activity, showed a good efficacy on methicillin resistant strains such as Gram-positive (*Staphylococcus* spp. and *Enterococcus* spp.) and Gram-negative (*Pseudomonas aeruginosa*, *Acinetobacter* spp., and *Klebsiella pneumoniae*) multidrug-resistant bacteria. However, other studies did not confirm the same efficacy of pracaxi oil on *Staphylococcus aureus* [127,141].

#### 4.6.5. Other Biological Activities

In a recent study, the Huh group [131] revealed through clinical trials and in vitro studies that pracaxi oil can lighten skin, reduce hyperpigmented lesions, and improve overall skin tone. These effects may result from modulation of melanogenesis and enhancement of the skin barrier function.

Otherwise, it was shown that pracaxi oil could be used as natural mask because of its behavior as a silicon-like material for cosmetic use [142].

## 5. Nanotechnology Applications of Amazonian Oils and Butters

Recent advances in nanotechnology have opened new perspectives for enhancing the bioavailability, stability, and efficacy of these natural ingredients.

This section explores the use of Amazonian oils and butters in nanostructured delivery systems, with a focus on their biological activities, based on recent scientific literature.

For example, nanoemulsions of açai seed oil have demonstrated a significantly higher antioxidant activity compared to the crude açai oil. Borges’ group [49] reported that nanoemulsified açai oil had higher concentrations of total phenolic and flavonoid compounds than the crude açai oil. Furthermore, the nanoemulsified oil outperformed the crude oil’s antioxidant capacity by more than 60% in both DPPH and ABTS measurements.

Beyond antioxidant potential, açai nanoemulsions were investigated in photodynamic therapy for melanoma, enabling enhanced delivery and penetration into tissues [52]. The Monge-Fuentes team [52] developed an açai oil-based nanoemulsion as a photosensitizer. The treatment led to an 85% reduction in melanoma cell viability in vitro, while sparing normal cells. In vivo tests on a mouse animal model showed 82% reduction in tumor volume after five photodynamic therapy sessions. The efficacy was thought to be linked to the high saturated fatty acid content of açai oil, which facilitates cellular membrane uptake, along with the cytotoxic effects of polyphenols.

The Coêlho group [99] suggested that bacuri butter, due to its high tripalmitin content, could serve as a functional lipid matrix for designing alternative drug delivery systems such as solid lipid nanoparticles and nanostructured lipid carriers. These systems are valuable for their ability to encapsulate both hydrophilic and lipophilic drugs, enhance stability, and offer controlled release properties.

Considering the wide spectrum of biologically active molecules of Amazonian oils, there is a strong potential for other future incorporation into nanocarriers for targeted delivery. As noted by the group of Benlloch-Tinoco [118], nanocarrier systems such as nanoemulsions and nanoparticles can protect polyphenols from degradation, improve their pharmacokinetics, and prolong their biological activity by avoiding first-pass metabolism.

The buriti oil showed good versatility in multiple applications ranging from emollient (cosmetics) to essential components (nanoemulsions and microencapsulation) [109,110]. However, because of its lipophilic character and tendency to oxidize, buriti oil is challenging to incorporate into water-based food products. To address this limitation, the Castro group [114] developed powder nanoparticles using porcine gelatin in combination with sodium alginate to encapsulate buriti oil. This formulation enabled water dispersibility while preserving, or even enhancing, the oil’s antimicrobial activity. Continued research on buriti oil, particularly in combination with complementary ingredients and emerging technologies, is crucial for emphasizing its full potential and maximizing its benefits, adding value and sustainability [110].

On the other hand, the cupuaçu seed extract was manufactured into nanocapsules with a chitosan (a biodegradable and biocompatible polysaccharide) coating presenting a moderate antimicrobial activity that acts as a penetration enhancer between the cells of the stratum corneum [117]. The in vitro study on skin explant emphasized that the cupuaçu chitosan-coated nanocapsules doubled the penetrated oleic acid in skin when compared to the uncoated nanocapsules [117].

However, we can assume that clinical trials in humans are essential to define therapeutic protocols and validate the efficacy of the nanomaterial containing Amazonian oils.

## 6. Conclusions

The aim of our review is to bring together research for the most important Amazonian oils, focusing on their lipids composition and proved biological activities. Also, we brought up some recent data on the specific composition of the fatty acids of the oils and oil’s biological activities and medical applications. We showed, for each oil, some specificities in fatty acids that could be related to biological activities. This review could be used as a basis to start new research to specifically show the mechanisms by which some of the metabolic pathways are activated by fatty acids of acai, andiroba, bacuri, buriti, cupuaçu, and pracaxi. If the synergy between certain fatty acids and the unsaponifiable matter (containing mostly lipophilic antioxidants) can be studied, much empirically studied healing knowledge used by the native population can now be decrypted.

The promising results of the scientific studies related in our review are encouraging for further clinical trials with Amazonian oils in various cosmetic and pharmaceutical applications.

Concerning the use of Amazonian oils in nanoparticles devices, we can retain, for example, the use of açai in nanoemulsion, cupuaçu in chitosan nanoparticles, buriti oil in alginate nanocapsules, and bacuri butter in solid nanoparticles, which does not permit yet a thorough critical assessment between studies. However, complementary studies on wound healing devices with nanoparticles containing Amazonian oils are currently under investigation.

## Figures and Tables

**Table 1:** Average content (%) of the most abundant fatty acids in some Amazonian oils and butters.

	Octonoic	Decanoic	Lauric	Myristic	Palmitic	Palmitoleic	Stearic	Oleic	Linoleic	Linolenic	Arachidic	Behenic	Lignoceric	Reference
	C8:0	C10:0	C12:0	C14:0	C16:0	C16:1	C18:0	C18:1	C18:2	C18:3	C20:0	C22:0	C24:0	
Açai oil *Euterpe oleracea*			2	1	25	4	2	57	9	1				[6]
Andiroba oil *Carapa guianensis*					27	1	9	52	9	2				[6]
Bacuri butter *Platonia insignis*			1	2	60	7	1	28	2					[6]
Buriti oil *Mauritia flexuosa*					17		2	75	5	1				[7]
Cupuaçu butter *Theobroma grandiflorum*					9		36	43	2		7	2		[7]
Pracaxi oil *Pentaclethra macroloba*					2		4	52	11		1	17	11	[6]

Shading criteria: □ < 15% < ■ < 50% < ■.

**Table 2:** Average content (%) of most abundant fatty acids in common vegetable oils and butters.

	Octonoic	Decanoic	Lauric	Myristic	Palmitic	Palmitoleic	Stearic	Oleic	Linoleic	Linolenic	Arachidic	Behenic	Lignoceric	Reference
	C8:0	C10:0	C12:0	C14:0	C16:0	C16:1	C18:0	C18:1	C18:2	C18:3	C20:0	C22:0	C24:0	
Almond oil *Prunus amygdalus dulcis*					7		2	67	23					[8]
Castor oil *Ricinus communis*					1	1	1	88 *	5					[9]
Coconut oil *Cocos nucifera*	6	6	47	19	9		3	7	2					[9]
Olive oil *Olea europaea*					11	1	2	75	10	1				[9]
Shea butter *Vitellaria paradoxa*					4		41	46	7		1			[10]
Sunflower oil *Helianthus annuus*					17		1	13	60		8			[8]

Shading criteria: □ < 15% < ■ < 50% < ■, * ricinoleic acid content.

**Table 3:** Observations on the biological activities of rare Amazonian oils.

Plant/Oil Origin	Type of Biological Activity	References
Açai *Euterpe oleracea* fruit oil	Antioxidant	[15,16,46,47,48,49]
	Anti-inflammatory Antinociceptive	[13,47,48,50]
	Antitumoral	[49,51,52]
	Antihypercholesterolemic	[53]
	Antimicrobial: *Staphylococcus aureus*	[54]
Andiroba *Carapa guianensis* seed oil	Antioxidant	[4,19,55]
	Anti-inflammatory	[4,5,22,25,55,56,57,58,59,60,61]
	Wound and tissue healing	[5,56,62,63]
	Antitumoral	[59,64,65,66,67]
	Anti-allergic	[68,69]
	Analgesic, anti-rheumatism, anti-arthritis	[70]
	Antimicrobial	[4,55,71,72,73,74]
	Antiparasitic	[4,22,71,75,76,77,78,79,80,81,82,83,84,85,86,87]
Bacuri *Platonia insignis* seed butter	Wound healing	[88,89,90]
	Cardioprotective	[91]
	Hypolipidemic	[91]
	Antimicrobial: anti-Leishmaniasis	[88,92,93]
Bacuri *Platonia insignis* seed extract	Antioxidant	[25,90,94,95,96,97,98,99,100]
	Anti-glycant	[27]
	Nitric oxide inhibitor	[27]
	Anti-inflammatory	[27,88,96,101]
	Anti-neurodegenerative: neuroprotective and central nervous system stimulator	[94,95,96,98]
	Anti-epileptic and anticonvulsant	[90,95,98,102]
	Gastroprotective	[103]
	Immunomodulatory	[104]
	Vasorelaxant	[26]
	Antiparasitic: anti-schistosomiasis	[90,96,100,105]
Buriti *Mauritia flexuosa* fruit oil	Antioxidant	[106,107,108,109,110,111]
	Anti-inflammatory	[112]
	Wound healing	[29,112]
	Photoprotective	[109,110,112,113]
	Antimicrobial activity: *Escherichia coli*, *Pseudomonas aeruginosa*, *Klebsiella pneumoniae*, *Staphylococcus aureus*	[106,107,112,114]
	Antifungal, antithrombotic, anti-proliferative, antidiabetic, prebiotic action	[29]
Buriti *Mauritia flexuosa* pulp powder	Estrogenic and antiandrogenic	[115]
Cupuaçu *Theobroma grandiflorum* seed butter	Wound healing	[116,117,118]
	Emollient effect	[36,119]
	Photoprotective	[118,120]
	Anti-neurodegenerative	[118,121]
Cupuaçu *Theobroma grandiflorum* seed extract	Antioxidant	[35,36,38,118,122,123]
	Antitumoral	[36,118]
	Antidiabetic	[118,123]
	Antimicrobial: *Plasmodium falciparum*	[38,124]
Pracaxi *Pentaclethra macroloba* seed oil	Antioxidant	[41,108,125]
	Anti-inflammatory	[43,126,127,128]
	Wound healing	[43,126,128,129]
	Against ulcers, stretch marks	[42,43]
	Antimicrobial	[130]
	Melanogenese regulation	[131]

## Data Availability

The authors from Dumato Ltd. [7] share, upon request, some results on the Amazonian oils shown in Table 1. However, all of Dumato’s research is not of public domain.
